# With Appreciation

**DOI:** 10.1002/ehf2.12232

**Published:** 2017-11-08

**Authors:** Monika Diek

1

We thank the following individuals who served as manuscript reviewers for the *ESC Heart Failure* Journal in 2017.

We highly appreciate their efforts. Fair, conscientious, and timely peer reviews contribute to the success of *ESC Heart Failure*.—The Editors

### *6 and more reviews*

Antonio Abbate
Ahmad Amin
Constantinos Bakogiannis
Patrick Doeblin
Athanassios Manginas
Thomas Meyer
Nasim Naderi
Iain Squire

### *3–5 reviews*

Manyoo Agarwal
Tolga Aksu
Hakan Altay
Siddhartha Angadi
Efstratios Apostolou
Dipanjan Banerjee
Elie Chammas
Francisco J. Cordova
Frauke Czepluch
Renato De Vecchis
Erwan Donal
Nicole Ebner
Sonja Hochmeister
Junichi Ishida
Attila Kertész
Masaaki Konishi
Steffen Kriechbaum
Goran Loncar
Thomas Marwick
Laura Meems
Serafim Nanas
Michel Noutsias
Julio Núñez
Line Lisbeth Olesen
Ambarish Pandey
Hitesh C. Patel
Simon Pearse
Pierpaolo Pellicori
Bertram Pitt
Eva Rye Rasmussen
Angelos Rigopoulos
Frances Russell
Parham Sadeghipour
Masakazu Saitoh
Karl Toischer
Tobias Trippel
Wesley Tucker
Dilek Ural
Farveh Vakilian
Jessica Webb

### *1–2 reviews*

Michaela Adamcova
Ligia M. Antunes‐Correa
Toshihisa Anzai
Isabelle Arnet
Hidetsugu Asanoi
Mohan Babu
Laura Varela Barca
Tuvia Ben Gal
Rodolfo Benatti
Kenneth Bilchick
Antoinette Blackman
Beáta Bódi
Vimalraj Boganashanmugam
Diana Bonderman
Claudio Borghi
Helle Bosselmann
Nadia Bouabdallaoui
Paco Bravo
Konstantinos Bronis
Leo Buckley
Monika Budnik
Federico Bussolino
Javed Butler
Alida Caforio
Richard Cheng
Wen‐Jin Cherng
Elizabeth Chuang
Andrew Clark
Lenard Conradi
Leslie Cooper
Tamás Csont
Éva Csősz
Seema Dangwal
Siamak Davani
Alexandre de Matos Soeiro
Renato De Vecchis
Torsten Doenst
Andre Duvinage
Mio Ebato
Frank Edelmann
Istvan Edes
Thomas Elgeti
Klaus Empen
Leif‐Christopher Engel
Stephan Ensminger
Justin Ezekowitz
Núria Farré
Stephan Felix
Dimitrios I. Fotiadis
Jennifer Franke
Lutz Frankenstein
Marcus Frany
Andrea Frustaci
Buntaro Fujita
László Fülöp
Angela Gallagher
Jens Garbade
Sergio García‐Blas
Georg Girke
Alexey Glukhov
Björn Goebel
Satish Govind
Damien Gruson
Hakan Güneş
Shashi Gupta
Tanush Gupta
Jong‐Won Ha
Karl Georg Haeusler
Camilla Hage
Nadjib Hammoudi
Veli‐Pekka Harjola
Christian Hengstenberg
Brian Hiestand
Berthold Hocher
Thomas Hofmarcher
Ignatios Ikonomidis
Ciro Indolfi
Victor Issa
Ewa Jankowska
Sheng Kang
David Kao
Oguz Karaca
Mahir Karakas
Nenad Karanovic
Seyed Ebrahim Kassaian
Tsutomu Kawai
David Kaye
Sebastian Kelle
Christoph Knosalla
Andras Komocsi
Naomi Kondo Nakagawa
Zsolt Kőszegi
Konstantinos Koutsampasopoulos
Recep Kurt
Valentina Kutyifa
Daniel Lenihan
Gerard Linssen
Oscar Lorenzo
Mark Luedde
Agnieszka Madej‐Pilarczyk
Martin Magnusson
Bernhard Maisch
Marcus Makowski
Alfonso Roberto Martiniello
Marios Matiakis
Sophie Mavrogeni
John J. McCarthy
Bruce McManus
Sven Meyer
Di Mauro Michele
Hendrik Milting
Veselin Mitrovic
Martin Moeckel
Selma Mohammed
Maria Luisa Morales
Oliver Mueller
Sanem Nalbantgil
Salomon Narodden
Kazuaki Negishi
Attila Nemes
Andrew Kei‐Yan Ng
Stuart A. Nicklin
Markku Nieminen
Maria Nikolaou
Mumin Noor
Mehmet Oezkur
Attila Oláh
Christian Opitz
H. Murat Ozdemir
Lorenzo Paladino
Patricia Palau
Andrea Passantino
Kanan Patel
Walter Paulus
Pierpaolo Pellicori
Renata Pereira
Roman Pfister
Massimo Piepoli
Ileana Pina
Gerhard Pölzl
Jordon Prutkin
Amresh Raina
Prabhakar Rajiah
Harsh Rawal
Andreas Rieth
Rebecca Ritchie
Howard Robertson
Miguel Rodríguez‐Santamarta
Gregory Roth
Tomasz Rywik
Yasuhiko Sakata
Maximilian Salcher
David Santer
Gianluigi Savarese
Renate Schnabel
Christian Schulze
Carsten Schwencke
Robert Sepp
Helen Sheriff
Victoria Simpkin
Christoph Sinning
Simon Stämpfli
Josef Stehlik
Stefan Stoerk
Gábor Szabó
Istvan Szokodi
Balaji Tamarappoo
W. H. Wilson Tang
Vinay Thohan
Stergios Tzikas
Kazutaka Ueda
Kairav Vakil
Bert Vandenberk
Ola Vedin
Chet R. Villa
Maurizio Volterrani
Tom Kai Ming Wang
Dirk Westermann
Jan Sebastian Wolter
Ali Yilmaz
Rosita Zakeri
Tanja Zeller
Shuyang Zhang
Michael R. Zile

